# Beneficial Effect of Wound Dressings Containing Silver and Silver Nanoparticles in Wound Healing—From Experimental Studies to Clinical Practice

**DOI:** 10.3390/life13010069

**Published:** 2022-12-26

**Authors:** Mateusz Rybka, Łukasz Mazurek, Marek Konop

**Affiliations:** Laboratory of Centre for Preclinical Research (CePT), Department of Experimental Physiology and Pathophysiology, Medical University of Warsaw, 02-106 Warsaw, Poland

**Keywords:** bacterial infection, silver nanoparticles, wound healing, wound dressing

## Abstract

Impaired wound healing affects hundreds of million people around the world; therefore, chronic wounds are a major problem not only for the patient, but also for already overloaded healthcare systems. Chronic wounds are always very susceptible to infections. Billions of dollars are spent to discover new antibiotics as quickly as possible; however, bacterial resistance against antibiotics is rising even faster. For this reason, a complete shift of the antibacterial treatment paradigm is necessary. The development of technology has allowed us to rediscover well-known agents presenting antimicrobial properties with a better outcome. In this context, silver nanoparticles are a promising candidate for use in such therapy. Silver has many useful properties that can be used in the treatment of chronic wounds, such as anti-bacterial, anti-inflammatory, and anti-oxidative properties. In the form of nanoparticles, silver agents can work even more effectively and can be more easily incorporated into various dressings. Silver-based dressings are already commercially available; however, innovative combinations are still being discovered and very promising results have been described. In this review article, the authors focused on describing experimental and clinical studies exploring dressings containing either silver or silver nanoparticles, the results of which have been published in recent years.

## 1. Introduction

Nanotechnology is a multidisciplinary scientific field that has drawn worldwide attention from various researchers in science and industry. It offers the facile synthesis of metal-based biocompatible nanomaterials that can be applied to a wide range of potential applications in medical and biological sciences, including medical diagnosis, medicine, health care, drug delivery, wound healing, the food industry, cosmetics, and environmental remediation. Non-healing wounds present a significant problem for both patients and healthcare systems. Wound infection is a complication of both acute and surgical wounds, with bacterial colonization and biofilm formation being present in the majority of nonhealing wounds. Therefore, successful wound healing often demands antimicrobial therapy. The high prevalence of infection together with the increasing risk of antibacterial resistance is the reason why potent alternatives to antibiotics are sought. In this context, biomaterials containing silver and silver nanoparticles are gaining attention in experimental and clinical trials as wound dressings. The use of silver-based compounds in wound care dates back to the early 1970s, which saw the introduction of the antimicrobial-antibiotic combination of silver sulphadiazine, providing an effective, broad-spectrum treatment [1]. The addition of silver in different forms increased the antimicrobial properties of basic dressings and protected against infections. In this review, we described the use of different materials containing silver or silver nanoparticles in preclinical and clinical settings.

## 2. A Short History of Silver in Medicine

The first descriptions of the use of silver in medicine come from antiquity. In Persia, it was noticed that drinking water that was stored in silver containers remained fresh for a longer time. In Egypt and Macedonia, silver plates were applied to wounds, as their positive effects on the wound healing process were noted [2,3]. Unfortunately, after antiquity, as with many medical disciplines, thousands of years passed before the next breakthrough moment that established the role of silver in medicine. In 1852, James Marion Sims [4] was the first to use silver wires as a suture. They were used to sew up a vesicovaginal fistula in a woman, for whom the previous 12 treatments using standard sutures were ineffective. At that time, Sims became the first renowned surgeon of American origin, and his introduction of silver sutures into surgery was one of the crucial discoveries of the 19th century in this field of medicine. The next period of common, widespread use of silver in medicine was in the time of World War I, when silver leaves were used to treat wounds in order to reduce the risk of infection and accelerate wound healing [5]. It should be mentioned that at the same time, Roe described the use of colloidal silver in the treatment of ophthalmic diseases [6]. Silver is routinely used to prevent ophthalmic diseases in newborns [7]. Although the empirical use of silver in human medicine has been known for thousands of years, the mechanisms of its antibacterial activity are complex and have been the subject of more recent studies.

## 3. Antimicrobial Properties of Silver and Silver Nanoparticles

**Figure 1:**
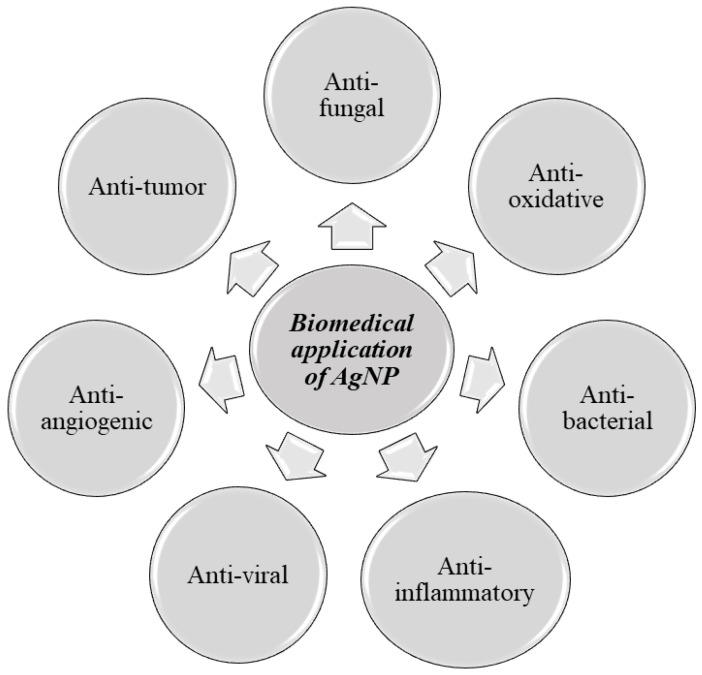
Biomedical application of AgNP.

In modern medicine, silver is often used in the form of silver nanoparticles (AgNPs) due to their physicochemical properties. In this form, silver still possesses its bioactive properties. Additionally, thanks to the size of nanoparticle, it can be used in a variety of situations. In medicine, AgNPs are usually applied as an antimicrobial agent. However, it is also worth noting that silver nanoparticles present anti-oxidative, anti-tumor, anti-angiogenic, and anti-inflammatory effects [8]. The anti-microbiological properties of AgNPs are closely related to the binding surface of the molecule, which makes smaller molecules like AgNPs exhibit greater antimicrobial properties [9]. Silver nanoparticles are effective both against gram-negative and gram-positive bacteria [10]. AgNPs also appear to be effective against such fungi as Aspergillus niger and Candida albicans, both alone—and when enhanced with substances like fluconazole [11,12]. The antiviral properties of AgNPs have been noticed in the studies of many viruses, including HIV, HBV, HSV, Influenza Virus, or SARS-CoV-2 [8,13]. A schematic diagram representing various applications of AgNPs is provided in Figure 1.

However, other nanoparticles with similar properties to silver are under investigation as well. For example, CuO and ZnO both present antibacterial properties. Like AgNPs, they can inhibit bacteria growth by creating reactive oxygen species. In 2022 Govindasamy et al. [14] tested a dressing containing ZnO, CuO and chitosan. Since chitosan can lead to the disruption of the cell membrane of bacteria it has a synergistic effect with ZnO/CuO nanocomposites, effectively killing both gram-positive and gram-negative bacteria. This is an interesting approach suggesting that the future of dressings containing AgNPs is to combine them with other nanocomposites of various metals to achieve a synergistic effect, using the entire range of AgNPs properties, such as antioxidant or anti-inflammatory effects. Other promising candidates that can be used in chronic wound therapy are antimicrobial peptides and selected herbs. The properties of AgNps, CuO/ZnO, Antimicrobial peptides, and herbs are listed in Table 1.

Unfortunately, often the use of silver in therapy is limited to superficial tissues, such as skin or conjunctival sacs. This is due to its uncertain effect on intestinal flora. Orally administered AgNPs seem to significantly change the composition of the intestinal microflora, which can affect the function of the whole organism [20]. Similar adverse effects were also observed for other nanomaterials such as CuNPs, Fe3O4NPs, CuONPs, and ZnNPs. The authors believe that this may limit their use in oral therapy in most cases, but further research is necessary, especially in the context of their use i.e., clostridium difficile infection (CDI), which is often characterized by disturbed gut microbiota. Studies show that nanoparticles can kill vegetative forms of Clostridioides difficile or Bacillus subtilis, while managing the toxins produced by these bacteria [21].

## 4. Mechanism of Action and Toxicity of Silver and Silver Nanoparticles

**Figure 2:**
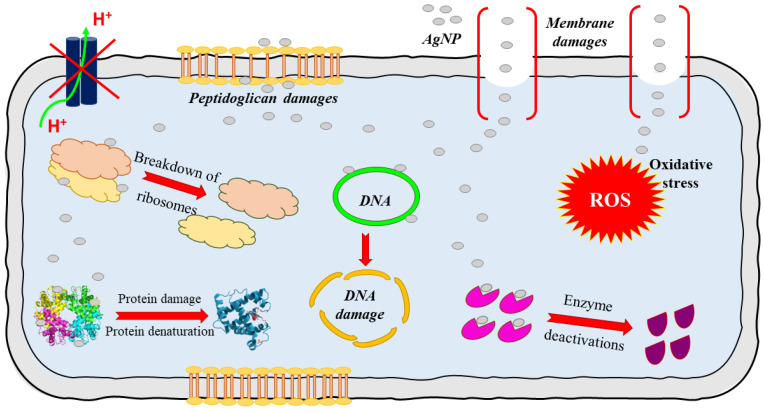
Mechanism of action of silver nanoparticles (AgNP).

The mechanisms by which silver nanoparticles act as antimicrobial agents are diverse and include: the formation of complexes with DNA and RNA, inactivation of enzymes and protein denaturation, ion exchange impairment, and shrinking and breaking of membranes (see Figure 2) [5]. Also, silver-killed bacteria present antibacterial activity—this unique mechanism is described as the “zombie” effect [22]. Silver nanoparticles possess microbiological properties due to their ability to constantly release silver cations. The silver cations act like a Lewis acid and willingly react with substances containing phosphate and sulphate residues, which are found in large amounts in cell membranes, proteins, ribosomes, and nucleic acids [22]. In this way, positively charged cell ions connect to the negatively charged surface of the cell membrane. This leads to structural changes in the membrane, shrinkage of the cytoplasm and finally the disruption of the cell wall [23].

The next mechanisms in which silver cations have an antibacterial effect are the result of their penetration into the cell. This can occur both through the influx of ions through the previously damaged cell membrane as well as through the porins in the outer membrane. The mutation of the genes encoding major porins is a confirmed mechanism of increased bacterial resistance to silver cations [24].

Once in the microorganism’s cell, silver cations damage it simultaneously in several mechanisms. These ions react with the disulfide bridges and thiol groups of proteins found in the mitochondria, ribosomes, and the cell membrane, resulting in the impairment of their functions. In addition, attaching silver to protein channels causes a change in their structure, resulting in a blockade of their function. This results in impaired transmembrane transport of potassium ions and thus limits the production of ATP in the cell [25]. Ag+ also disrupts the function of the respiratory chain. This leads to an additional increase in the number of reactive oxygen species in the cytoplasm of the cell [23].

**Figure 3:**
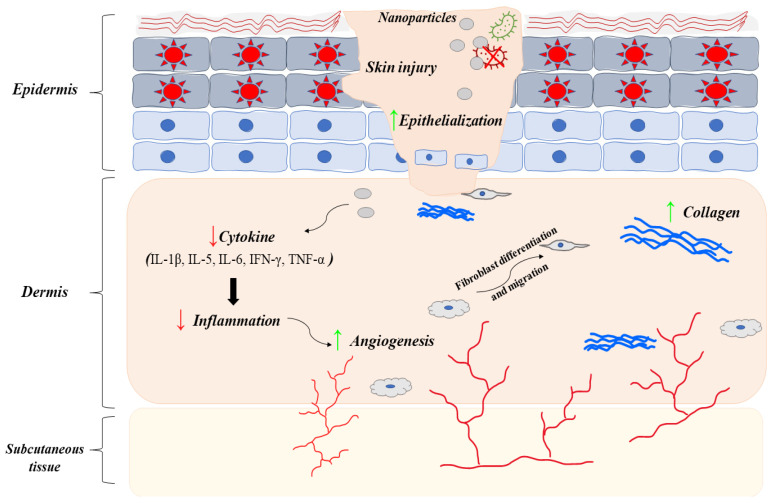
The healing potential of nanoparticles at the skin injury (↓—decrease, ↑—in-crease). AgNPs are associated with cytokine modulation, which leads to a reduction in inflammation, innate immune response, and scar formation, as well as the differentiation and migration of keratinocytes. AuNPs have also demonstrated anti-inflammatory properties, as well as increasing epithelization and collagen deposition. They can also modulate proteins involved in the healing process, such as superoxide dismutase, interleukin-8, interleukin-12, TNF-α, VEGF, and angiopoietin. Other nanoparticles, including copper, and zinc oxide, have shown similar effects, while they also have antimicrobial properties and increase angiogenesis.

Silver is known to have a strong antibacterial effect by damaging the DNA in the cell of the microorganism. This is caused both by the reaction of silver ions with nucleic acids and by the damage that is secondary to the oxidative stress caused by the influx of silver cations into the cell. In the first mechanism, silver cations directly react with nucleic acids, damage them, and cause various types of mutations, including those in key DNA repair genes [23]. In addition, silver, by forming complexes with DNA, inhibits the proper transcription process and prevents bacteria from dividing and multiplying [25]. Additionally, Ag+ damages DNA strands by increasing the level of oxidative stress in the cell. Oxidative stress increases simultaneously in two mechanisms of successive reactions. First, silver cations catalyze reactions that lead to the formation of reactive oxygen species (ROS) in the cell. In addition, silver binds to reactive oxygen species scavengers, decrease their activity and increase oxidative stress. One of the basic molecules involved in scavenging free radicals is glutathione (GSH). Once inside the cell, silver ions transform glutathione (GSH) into its inactive, oxidized form (GSSG) [25,26]. Silver, by interrupting antioxidant mechanisms at many stages, minimizes the chances of the development of bacterial resistance mechanisms to oxidative stress. In addition, the created free radicals damage mitochondria, denature enzymes and cause lipid peroxidation [27,28]. It has been shown that the amount of newly generated ROS is proportional to the concentration and duration of action of AgNPs [29]. There are also several studies suggesting that silver nanoparticles possess immunomodulatory properties, which may enhance their antibacterial effect by coordinating inflammatory processes (Figure 3) [30,31]. In vitro studies showed that AgNPs may decrease the production of IL-1β, IL-5, IL-6, IFN-γ, and TNF-α [32,33]. Anti-inflammatory mechanisms were also described after administering silver to burn wounds and in the mouse model of IBS [34,35].

Despite the above-mentioned properties, silver nanoparticles also have anti-angiogenic properties and promote collagen deposition. The inhibition of angiogenesis occurs as AgNPs have an influence on several factors [36]. One of the mechanisms by which silver nanoparticles regulate angiogenesis is due to the effect on the PI3K/AKT signaling pathway. It has been confirmed in several studies that AgNPs suppresses the survival and proliferation of endothelial cells through the downregulation of PI3K/AKT signaling pathway [37]. In addition, silver nanoparticles can inhibit angiogenesis induced by FGF-2, which was confirmed both in the CAM (Chick cho-rioallantoic membrane assay) model and in the FGF-2 mouse matrigel model [38]. The third mechanism was described by Yang et al. and it is associated with the inhibition of HIF-1 activation, responsible for the formation of new vessels in response to hypoxia of the surrounding tissues [39]. Despite the anti-angiogenic properties of AgNPs in vitro studies, sometimes biomaterials coated with AgNPs can promote angiogenesis in chronic wound healing. However, it usually happens as a result of an anti-inflammatory effect of silver-coated dressings leading to the acceleration of wound healing [40]. In addition to the previously mentioned AgNPs properties, silver also affects wound healing through increased collagen deposition. Studies made by Lin et al. showed that silver-containing ACF increases the proliferation of fibroblasts within the skin [41]. It has also been proven that prolonged exposure of epithelial cells to silver nanoparticles stimulates them to secrete TGF-β [42]. Both the greater number of fibroblasts, as well as their stimulation by TGF-beta, result in increased production and deposition of extracellular matrix proteins, such as collagen [43]. In recent years, a lot of research has been conducted on oxidants, antioxidants and oxidative stress. As it was mentioned before, silver nanoparticles increase oxidative stress in bacterial cells, and this is one of the many mechanisms in which it exhibits antibacterial activity. However, it was noted that depending on the extract from which the AgNPs were obtained, they can reduce oxidative stress in healthy cells. Mohanta et al. [44] showed that silver nanoparticles synthesized from the leaves of Erythrina suberosa, have antioxidant properties and increase the ability of AgNPs to scavenge free radicals by DPPH. In addition, for AgNPs obtained from Cestrum nocturnum, higher antioxidant activity was confirmed compared to vitamin C, while maintaining strong antibacterial properties [45].

**Figure 4:**
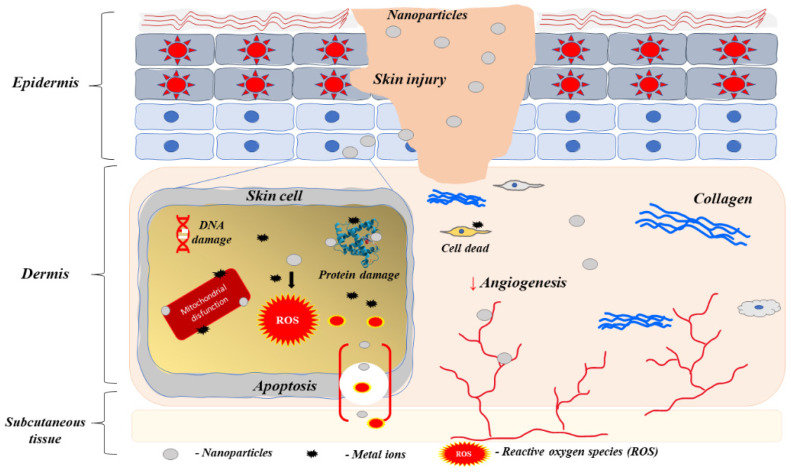
Nanoparticles toxicity at the site of injury (↓—decrease, ↑—increase). It has been shown that nanoparticles damage skin cells such as keratinocytes and fibroblasts by inducing ROS after entry through endocytosis, causing mitochondrial damage, genetic toxicity, and lipid peroxidation. Similar damage can also occur to other cells, as well as protein damage, changes in signaling and metabolism, reduced attachment, and migration and induced cell death.

Of course, all these mechanisms in which AgNPs damage bacterial cells will have an impact on human cells as well (Figure 4). The toxicity of silver will depend both on its form and the route of administration. Silver ions are characterized by the greatest toxicity among all silver forms, so administering them as a silver salt will be more toxic when compared to the AgNPs [46]. In addition, the skin is a better mechanical barrier against silver than mucous membranes or skin wounds [47]. This seems to be confirmed by the results of studies, that say that exposure of the mucosa of the respiratory or the gastrointestinal tract to silver ions more often leads to side effects when compared to skin application. Accumulating silver in the body causes a disease called argyria. Argyria is characterized by a change of the color of the skin to blue-grey. Moreover, the color change of the conjunctiva may be present. Argyria is a dermatological manifestation of the accumulation of silver in the human body, therefore no symptoms from other organs are present in this disease, however silver accumulation itself can be fatal [48]. If argyria appears in its generalized form, the recovery takes many years after the end of the exposure to silver. Silver is eliminated from the body with urine and bile, so renal and hepatic failure may also lead to the accumulation of silver in the body [49]. Less common side effects of silver treatment include renal and hepatic insufficiency and hematological diseases [50]. Other adverse reactions are related to the route of the administration of silver. Cases are describing respiratory insufficiency, but usually it is just a mild respiratory irritation. Abdominal pain is a common adverse effect when silver is taken orally, however it can also appear when silver is administered by different routes. It should be noted that the overall incidence of all of these side effects is very low. Yet, in order to limit the amount of silver administered to the body, it is usually used with a substance that presents a synergistic effect. Thus, silver is being used with norfloxacin or streptomycin to enhance the antibacterial effect or together with fluconazole to enhance the antifungal properties [51,52]. AgNPs can also be combined with substances that present immunomodulatory effects and accelerate the overall wound healing process [53].

## 5. Biomaterials Containing Silver and Silver Nanoparticles—Experimental Studies

Biomaterials are used in regenerative medicine as wound dressings, implantable materials, controlled-release carriers, or scaffolds for tissue engineering. Scientists around the world used different biomaterials (like keratin, cellulose, chitosan, and polymers) supplemented with silver or silver nanoparticles [53,54,55]. Regardless of the type, dressings are being tested on various wound healing models in laboratory animals (Table 2). However, chronic, nonhealing wounds are a serious problem for people suffering from diabetes. Therefore, various scientific groups have studied potential dressings based on natural or synthetic substances in healthy and diabetic conditions. which would significantly accelerate the healing process (Table 3).

Niyas Ahamed et al. [55] examined composites containing regenerated cellulose (RC) and chitosan (Ch) impregnated with silver nanoparticles (AgNP) with and without gentamicin (G) as a wound dressing in healthy Wistar rats. They showed that complete wound closure was observed on day 25 in animals treated with RC-Ch-Ag and RC-Ch-Ag-G, and over 30 days in control wounds. There was no significant difference in the healing pattern between the RC-Ch-Ag and RC-Ch-Ag-G dressing materials, and the presence of the antibiotic only reduced the number of inflammatory cells. In conclusion, these observations indicate that RC-Ch-Ag and RC-Ch-Ag-G composites can be tried as dressing materials in clinical wounds, but future studies in animal models are needed before application in humans.

Qian et al. [40] fabricated an asymmetric wettable dressing based on chitosan (CTS), silk fibroin (SF), and stearic acid (SA) with a composite of exosomes and silver nanoparticles (CTS-SF/SA/Ag-Exo dressing) for promoting angiogenesis, nerve repair and infected wound healing in BALB/c male mice. In vitro characterization showed that CTS-SF/SA/Ag-Exo dressings possessed multifunctional properties including broad-spectrum antimicrobial activity and promoted the proliferation of human fibroblasts. Moreover, it’s retaining moisture and maintains the electrolyte balance. In vivo examination showed that the CTS-SF/SA/Ag-Exo dressing enhanced wound healing by accelerating collagen deposition, angiogenesis, and nerve repair in a *P. aeruginosa* infected wound.

Mohseni et al. [56] examined porous polycaprolactone (PCL)/polyvinyl alcohol (PVA) nanofibers loaded with different concentrations of silver sulfadiazine (SSD) or silver nanoparticles (AgNPs) and compared comprehensively in vitro and in vivo in full-thickness skin wound model in male Wistar rats. In vitro examination showed that SSD and AgNPs indicated a strong and equal antimicrobial activity against *S. aureus*. They observed that SSD had more toxicity against fibroblast cells over one week of incubation. An in vivo examination showed that the obtained dressings indicated sufficient flexibility and hydrophilicity, which resulted in an adequate adhesion to the wound closure. In the control group (without any treatment) after 30 days the wound were healed on 31%, while the group treated with PCL/PVA (without antimicrobial components) indicated 44% wound closure. Moreover, the presence of antimicrobial components in the PCL/PVA nanofibers resulted in a lower inflammation response leading to faster proliferation and maturation phases. Due to the higher biocompatibility of AgNPs than SSD, faster angiogenesis, epithelialization, and subsequently, remodeling was observed for the wound dressings loaded with AgNPs. The group treated with the highest concentration of AgNPs (10wt%) showed the fastest healing process leading to a final epithelialization with 96% wound closure after 30 days. This study suggested that PCL/PVA-AgNPs have higher biocompatibility and regulate the wound healing process more efficiently compared to PCL/PVA-SSD.

Tra Thanh et al. [57] examined multi-coated electrospun polycaprolactone/gelatin/nanosilver (EsPCLGelAg) membranes as a wound dressing in mice. The obtained biomaterials were tested for their antimicrobial properties against *P. aeruginosa* and *S. aureus*. The coated membranes showed higher antimicrobial activity compared to the single-coated membranes. In vivo results showed that the six-time coated EsPCLGelAg membrane (S6) accelerated the wound healing rate. Moreover, multicoated membranes did not stick to the wound when peeling off, which prevented damage to the newly formed granule tissue compared to the monocoating tissue.

### Diabetic Conditions

In the last few years, AgNPs gain the most insight into the medical field due to their excellent chemical stability, high conductivity, catalytic activity and localized surface plasma resonance [58,59]. AgNPs can also be used as an intrinsic therapeutic approach as silver is known to produce antibacterial and anti-inflammatory properties, which promote the healing of chronic wounds.

Konop et al. [53] described the application of keratin-silver nanoparticle biomaterials as a wound dressing in full-thickness skin wound healing in diabetic mice. In vitro examination showed that obtained dressings inhibited the growth of *E. coli* and *S. aureus*. They showed that wounds treated with keratin dressing supplemented with AgNP healed significantly faster than untreated. They also did not observe the toxic effect in vivo. In another study, they showed that silver is slowly released from the dressing compared with commercially available dressings Atrauman Ag and Aquacel Ag [60].

Reddy et al. [61] examined the wound healing capabilities of gallocatechin (GC) and silver nanoparticles (AgNPs) impregnated patches in diabetic rats. They showed, that wound healing rate in diabetic rats dressed with the 13.06μM GC-AgNPs’ cotton gauze patch (CGP2) and 26.12 μM GC-AgNPs cotton gauze patch (CGP3) & silver sulfadiazine (AgS) patches were higher compared to control group. In addition, apoptosis markers such as caspase-3, caspase-9 and Bax levels were decreased, while the anti-apoptosis marker (Bcl-2) and proliferation marker (PCNA) levels were elevated in diabetic rats dressed with CGP2 and CGP3, compared to diabetic rats dressed with DC + CGP1. In conclusion, the results showed that GC-AgNPs-CGP (CGP2 & CGP3) dressing on rats with diabetic wounds reduced changes in the Wnt3a/β-catenin pathway, resulting in less apoptosis and more proliferation, thus improving diabetic wound healing.

Kaur et al. [62] testes nano-insulin formulation in non-diabetic and diabetic rats. Each group was next divided into four sub-groups including control, AgNPs, ATE-Insulin and IAgNPs treatment with six rats in each. They showed that insulin-loaded AgNPs (IAgNPs) significant improvement in healing activity in vitro (HEKa cells) and in vivo (Wister Rats) in comparison with the control in both normal and diabetic conditions. Histological evaluations on the 5th and 11th day showed a significant decrease in the level of leukocyte infiltration by IAgNPs treatment compared to controls and other treatment groups. Also, in the IAgNPs treated groups, faster deposition of collagens and rapid re-epithelization were observed in comparison with other sub-groups or controls. Moreover, a rapid decrease of pro-inflammatory (IL-6, TNFα) cytokines and an increase in anti-inflammatory (IL-10) cytokines were observed in IAgNPs treated animals.

Masood et al. [63] examined chitosan polyethylene glycol (PEG) hydrogel-impregnated silver nanoparticles as a wound dressing in diabetic rabbits. In vivo experiments showed a higher porosity, higher degree of swelling and higher water vapor transition rate (WVTR) for silver nanoparticle-impregnated hydrogel compared to bare chitosan-PEG hydrogel as well as improved antimicrobial and antioxidant properties. In vivo experiments showed that wounds treated with AgNPs incorporated chitosan-PEG hydrogel healed significantly faster than positive control (32.9 ± 2.1%), AgNPs (26.9 ± 1.4%) and plain CS/PEG (43.5 ± 2.2%) formulations. Also, AgNPs incorporated chitosan-PEG hydrogel showed scar formation with significant wound contraction and better re-epithelialization and significant keratinocytes migration as compared to plain chitosan hydrogel where wounds showed scar and scab formation with less wound contraction. Summarizing, faster healing of wounds in vivo experiments with chitosan-PEG hydrogels impregnated with AgNP as compared to bare chitosan-PEG hydrogels lead to the conclusion that these wound dressing can be applied later for treating wounds in human subjects.

Singla et al. [64] examined a nanocomposite dressing consisting of plant cellulose nanocrystals (CNCs) impregnated by an optimized concentration of AgNPs in Swiss albino diabetic mice. The topical application of fabricated nanocomposite as strips and ointments on diabetic mice showed improved tissue repair (~99% wound closure) by increasing the angiogenesis and collagen deposition and accelerating the re-epithelialization. The synergism of CNCs and AgNPs resulted in higher expression of growth factors (PDGF, VEGF), while lower expression of pro-inflammatory factors (IL-6, TNF-α). The achieved results suggest the potential of the developed antibacterial, cyto-compatible and nanoporous NCs with optimized concentrations of AgNPs as ideal dressings for effective wound treatment.

Zhao et al. [65] examined a conductive hydrogel fabricated from a supramolecular assembly of polydopamine decorated silver nanoparticles (PDA@Ag NPs), polyaniline, and polyvinyl alcohol, namely PDA@Ag NPs/CPHs as a potential wound dressing in male Sprague–Dawley diabetic rats. Remarkably, PDA@Ag NPs/CPHs exhibit broad antibacterial activity against gram-negative and gram-positive bacteria. In vivo experiments showed that PDA@Ag NPs/CPHs have a significant therapeutic effect on diabetic foot wounds by promoting angiogenesis, accelerating collagen deposition, inhibiting bacterial growth, and controlling wound infection. To the best of the authors’ knowledge, this is the first time that conductive hydrogels with antibacterial ability are developed for use as epidermal sensors and diabetic foot wound dressing.

Shi et al. [66] prepared hybrid hydrogels with maleic acid-grafted dextran and thiolated chitosan and utilized it as a wound dressing to accelerate wound healing in diabetic Sprague-Dawley (SD) rats. In vitro examination showed that NIH 3T3 cell viability of AgNPs@CNDM was higher than that of AgNPs and indicating that AgNPs@CNDM could decrease the cytotoxicity of AgNPs. Moreover, they showed that silver-containing hydrogels provided a slow and sustained Ag+ release. The porous structure of the dressing could provide a humid microenvironment and absorb the exudation from the wound. In vivo experiments showed that wounds treated with examined hydrogels heald significantly faster compared with the AgNP and the CNDM-treated group. The upregulation of CD68+ and CD3+ expression levels and histological examination confirmed that the AgNPs@CNDM hydrogel could trigger immune responses in the treatment of wound healing. Summarizing, the obtained results show that this antifouling hybrid hydrogel wound dressing provided a promising strategy for the treatment of diabetic ulcers.

Gaikwad et al. [67] conducted a study on Wistar rats, where they examined the effective-ness of mycosynthesized silver nanogel in the incision, excision, and burn wound–healing model. Three groups received gels with different concentrations of AgNPs, the fourth group received a commercial dressing, the fifth received the gel dressing alone, and the sixth group was a negative control. Promising results of dressings containing AgNPs have been obtained for all types of wounds. Nanogels incorporated with 0.5 mg/g AgNPs showed superior tensile strength (380 g vs. 213 g in control group) not only compared to other wounds treated with experimental dressing but also compared to commercially available dressings. Excision wounds treated with a commercial dressing were characterized by increased wound contraction, especially on the 15th and 20th day of the experiment (98.3% vs. 85.77%). A similar phenomenon has been observed for burn wounds (98.60% vs. 85.48%).

To reduce the side effects of silver and other antimicrobial substances, scientists are trying to combine them with substances that have a synergistic effect. Banna et al. [68] presented results of the study in which a gel containing both neomycin and AgNPs was tested in the treatment of wounds in rats. 45 rats were divided in such a way that some of the animals were treated with a gel containing only Ag+, some only with neomycin, and some with a dressing containing both neomycin and AgNPs. Both in the groups receiving only AgNPs and neomycin alone, accelerated wound healing was observed, however, the best effects were observed for the experimental dressing containing both neomycin and AgNPs, and the effect was found to be dose dependent. On day 15, wounds treated with neomycin silver nano-composite gel were healed in 95.3% while wounds in the untreated group were healed only in 66.8% of the initial area.

Another antibiotic that was tested for its synergistic effect with silver is colistin. Wali et al. [69] explored the effectivity of the dressing made of human amniotic membrane (hAM) that was impregnated with colistin and AgNPs in rats with infected burn wounds. The wounds were inoculated with MDR strains of *P. aeruginosa* and *K. pneuomoniae* and rats were divided into five groups with negative control treated with distilled water-dHAM, positive control that left without any treatment, and three different observation groups where either colistin-dHAM, AgNPs-dHAM or colistin + AgNPs-dHAM was used. Results showed that colistin + AgNPs-dHAM was the most effective in the healing of infected wound burns (with 87.4% wound contraction infected *P. aeruginosa* vs. 41.3% in positive control and 92.0% against K. pneumoniae vs. 64.7% in positive control).

An inflammatory process can impair the antibacterial effect of the tested substance. Kong et al. [70] decided to test riclin-capped silver nanoparticles for their an-ti-inflammatory as well as antibacterial properties. The experimental dressing was applied to male mice. Riclin/AgNPs dressing was found to be useful not only in the treatment of S. aureus infection but also decreased mRNA levels of IL-1B, IL-6 and TNF-alpha when compared to untreated and riclin treated wounds, proving the antiinflammatory properties of attaching AgNPs to various dressings.

There are many commercially available dressings that are containing silver combined with other substances. Carvalho et al. [71] conducted a study in which they compared many different commercially available dressings containing silver nanoparticles for the treatment of patial-thickness burns. Rats were divided into six groups, one received 0.9% NaCl, one received dressing containing 1% silver sulfadiazine, and the other four groups were treated with either Silvercel, Mepilex Ag, Aquacel Ag or Acticoat. All dressings containing silver turned out to be more effective than the NaCl used in the control group. Different dressings were effective at various stages of the wound healing process, however, Silvercel and Acticoat turned out to be the most effective.

Davis et al. [72] conducted a study using a porcine model in which they examined the effectiveness of anti-biofilm silver hydrofiber (ABSH) dressings in the treatment of deep-partial thickness wounds. This is a new generation of dressings containing silver with proven effectiveness in reducing the total mass of biofilm within the wound. Biofilm is confirmed independent risk factor of delayed wound healing [73], therefore development of this type of dressings seems reasonable. In this study, the control wounds were treated with dressings with proven effectiveness in the treatment of wounds, such as silver hydrofibre and polyurethane foil when the observation wounds were treated with ABSH. After eight days there was no statistical difference in the healing duration of deep-partial thickness wounds between those three groups.

One of the natural compounds with promising properties to combine with silver is curcumin, as it possesses many health benefits similar to AgNPs such as antioxidant, anti-inflammatory, and anti-microbial properties. Liu et al. [74] produced the dressing based on the combination of electrospun chitosan nanofibers and silver@curcumin nanoparticles. In vitro studies showed that silver and curcumin possess synergistic effects on the antibacterial activity as they were more effective against *P. aeruginosa*, *S. aureus*, and *E. coli* than commercially available silver dressings and chitosan nanofibers. In vivo studies showed accelerated wound contraction in the observation group at all time points. On the day 12 wound treated with silver@curcumin dressing was almost completely healed while the control wound was contracted in approximately 70%.

**Table 2:** Summarizing of application of silver impregnated wound dressing in an experimental wound model.

Author	Healthy or Diabetic	Type of Wound Dressing	Healing Rate (*p*-Value)	Wound Status	
				Control Wound	Dressed Wound
Experimental Studies					
Niyas Ahamed et al. [55]	Healthy	Composite	*p* > 0.05	Regenerated cellulose + chitosan + AgNPs	Regenerated cellulose + chitosan + AgNPs + Gentamicin
Qian et al. [40]	Healthy	Asymmetric wettable dressing	*p* < 0.05	Gauze	Dressing based on chitosan, silk fibroin, stearic acid with a composite of exosomes and AgNPs
Mohseni et al. [56]	Healthy	Porous nanofibers	*p* < 0.05	No dressing	Polycaprolactone/polyvinyl alcohol + AgNPs
Tra Thanh et al. [57]	Healthy	Multi-coated membranes	*p* < 0.05	No dressing	Electrospun polycarpo-prolactone + gelatin + nanosilver
Konop et al. [53]	Diabetic	Fur keratin-derived powder	*p* < 0.05	No dressing	Fur keratin-derived powder containing AgNPs (FKDP + AgNP)
Reddy et al. [61]	Diabetic	Cotton gauze Patches	*p* < 0.05	Cotton gauze	Cotton gauze patches + gallocatechin and AgNPs
Kaur et al. [62]	Healthy and Diabetic	Different nano-formulations + gel	*p* < 0.05	Saline + gel	AgNPs/ATE-Insulin/IAgNPs + gel
Masood et al. [63]	Diabetic	Hydrogel	*p* < 0.05	No dressing	Chitosan-PEG + AgNP
Singla et al. [64]	Diabetic	Nano-composite dressing	*p* < 0.05	Vaseline	Cellulose nanocrystals + AgNPs
Zhao et al. [65]	Diabetic	Hydrogel	*p* < 0.05	Phosphate Buffered saline	Polydopamine decorated silver nanoparticles, polyaniline and polyvinyl alcochol
Shi et al. [66]	Diabetic	Hydrogel	*p* < 0.05	No dressing	Maleic acid-graften dextrant and thiolated chitosan + AgNPs
Gaikwad et al. [67]	Healthy	Hydrogel	*p* < 0.05	No dressing	Mycosynthesized Silver Nanogel
Banna et al. [68]	Healthy	Nano-composite gel	*p* < 0.05	No dressing	Silver nano-composite gel
Wali et al. [69]	Healthy	Human amniotic membrane	*p* < 0.05	No dressing	Colistin and AgNPs impregnated amniotic membrane
Kong et al. [70]	Healthy	Nanocomposite gel	*p* < 0.05	No dressing	Polysaccharide riclin and AgNPs
Carvalho et al. [71]	Healthy	Different silver-based dressings	*p* < 0.05	No dressing	Silvercel, Mepilex Ag, Aquacel Ag, Acticoat
Davis et al. [72]	Healthy	Hydrofiber	*p* < 0.05	Polyurethane film	Anti-biofilm silver Hydrofiber
Liu et al. [74]	Healthy	Nanofiber	*p* < 0.05	No dressing	Silver@curcumin and electrospun chitosan nanofibers

**Table 3:** Summarizing of application of silver impregnated wound dressing in an experimental wound model.

Type of Wound Dressing	Advantages	Disadvantages	References
Hydrocolloid	Absorbs exudate and maintains a moist environment. Thermal isolation for the wound. Pain relief properties. Easy to use in therapy	Contraindicated in infected wounds with associated clinical symptoms. The lack of experience in changing this dressing, easy to damage the skin and surrounding tissues.	[75,76,77,78]
Hydrogel	Maintains a moist wound healing environment, Ability to absorb wound exudate, gas permeable	Often requires a second layer of dressing, poor mechanical properties, some require frequent moistening to maintain properties	[75,76,77]
Scaffolds	Absorbs exudate, low adherence to the skin, biocompatible, biodegradable, gas permeable, support cell growth	Secondary dressing is necessary	[79]
Gauze	Cheap, easily available. The main function is to protect the wound from the external environment, easy to use, clinicians have extensive experience with this type of dressings	Sticks to the wound, make it difficult to change dressings. Does not provide moisture environment. No significant effect on wound healing.	[75,77]
Alginate	Absorbent, non-stick. Controlled release of substances with which they were enriched.	Contraindicated in dry wounds, often cost of therapy	[75,76,77,78]
Composite	Have the properties of two or more different dressings that were combined to create composite dressing. Easy to apply, provides a moist environment.	High cost of therapy	[75]

## 6. Clinical Application of Wound Dressings Containing Silver and Silver Nanoparticles

Promising results received from experimental studies confirming the safety and efficacy of silver nanoparticle dressings quickly led to a series of clinical trials [80,81]. These studies provided useful data that allowed several dressings to be brought to market. They are now being used in diseases such as venous ulcers, diabetic foot, burns, or surgical wounds [82].

However, several years after the first dressing of this type was introduced to the market, the potential of silver nanoparticles in wound healing has not been fully exploited, and it was necessary to conduct further research on AgNP dressings. Increasing the analgesic and anti-inflammatory properties of the invented dressings is necessary, as silver does not possess it at a sufficient level. That is why various types of natural-derived biomaterials were enriched with silver to find the perfect dressing presenting all necessary properties (Table 4).

In 2017, Meekul et al. [83] conducted a clinical trial in which they tested the effectiveness of alginate silver dressing in patients suffering from necrotizing fasciitis wounds. Four outcomes were investigated in the study: wound bed preparation, time spent in the hospital, experienced pain, and total cost of hospital stay. The average wound bed preparation time was 10 days shorter for the group treated with Ag-dressing when compared with the group receiving standard treatment (21.39 vs. 31.87 days). Moreover, the time of hospitalization itself was shorter (20.99 vs. 29.19 days). Importantly, the pain score in patients treated with alginate silver dressing was significantly lower than in the control group, which is a struggle among silver-based dressings available commercially.

Hahn et al. [84] decided to investigate the effect of silver-impregnated negative-pressure dressings on the number of bacteria in open wounds of the lower extremities of patients. Participants were divided into two groups, the control group received conventional negative-pressure wound therapy (NPWT + polyurethane foam) while the research group received negative-pressure wound therapy, but the polyurethane foam was additionally coated with silver. Results showed that bacterial growth was lower in the silver-treated group and that the difference between groups increased over time. This conclusion was confirmed both for bacteria taken from the wound surface and tissue culture.

In 2021, JiHui Chen’s team conducted an experiment in which they examined the effectiveness of using silver-enriched dressings in the treatment of wounds in patients with pemphigus vulgaris (PV) [85]. A total of 28 patients in the study group received a dressing containing silver, while 30 patients in the control group received gauze soaked in 0.5% povidone iodine solution. The mean duration of wound healing in the Ag-group was significantly shorter (43.72 vs. 55.00 days). The hospitalization time was also shortened (33.72 vs. 43.64 days) and the frequency of applied dressing changes was reduced (average 18 dressings vs. 43.5). Although no statistical significance was achieved in the incidence of infection, it is also worth noting that it was lower in the research group (1 vs. 3, *p* = 0.302). It is important, as infection and sepsis are the main cause of mortality in the group of patients suffering from PV [86].

Matilda Karlsson et al. [87] decided to conduct a prospective study that compared two treatment methods in children with partial thickness scalds. 58 children were divided into two groups, one received porcine xenografts and the other one was treated with a silver-foam dressing. The primary outcome was the healing time, while secondary outcomes were for example pain, the necessity of surgery, infection of the wound, hospitalization time and the frequency of the dressing change. Wounds covered with silver-based dressings showed significantly shorter healing time (15 days vs. 20.5 days). This difference was seen in all wounds, even those that were covering more than 20% of the total body surface area. All the other outcomes were found to be similar between the groups, except for the number and time of dressing changes, which again fared better for the group treated with silver foam.

In 2022, Akin et al. [88] published the results of their study in which they tested the effectiveness of a silver dressing in reducing the risk of surgical site infection in patients after ostomy closure. It is a well-known fact, that gastrointestinal tract is often colonized by multidrug-resistant strains of bacteria and that’s why surgical site infections after colorectal surgeries can be hard to cure. 31 patients that were enrolled in this study—15 of them were treated with conventional gauze dressings for 1–2 days after the ostomy was closed. After that wound was cleaned with the 10% povidone iodine, the gauze dressings were applied and then there were changed daily for 5 days. The wounds of patients from the study group were treated with a dressing containing silver. The dressing was applied in the operating room and was not changed for 5 days after the procedure. In the 30 days, it was observed that surgical site infection occurred in 26.7% of patients in the control group, while no patient from the study group developed a surgical site infection.

To check the effectiveness of silver nanoparticle dressings, Asgari and his team [89] conducted a study in which they explored their effectivity in the treatment of pressure ulcers with spinal cord injuries. 70 patients suffering from spinal cord injury were enrolled in the study and then divided into two equal groups, one was receiving a hydrocolloid dressing and the other an AgNPs-based dressing. The Bates-Jensen Wound Assessment Tool was used to assess the effectiveness of the dressings at four-time control points. It was noticed that ulcers healed effectively under the influence of both therapies. The relative reduction of the BWAT score was greater for Silver nanoparticles, but the score was not statistically significant.

It is also worth mentioning the study that checks the effectiveness of silver-based dressings in acute wounds. Berard et al. [90] conducted a pilot study involving 32 patients. 16 of them received minimal adherent silver dressings (MASD), while the remaining 16 received standard dressings (SoC). There was no difference in the duration of wound healing between the two groups. However, acute wounds tend to heal effectively even in the absence of any dressing assistance, making it difficult to achieve an effect on wound duration rate. However, wounds treated with mini-adherent silver-containing dressings were characterized by less pain experienced by the patient (SoC pain score was 2.8 vs. MASD 1.1, *p* < 0.05), especially during dressing changes.

Although acute wounds heal quickly and effectively, can leave scars that stay for the rest of patients’ lives. Miner et al. [91] decided to investigate whether a hydrogel containing silver particles can not only accelerate the healing of a postoperative wound, but also decrease the probability of scar formation. 40 patients with foot and ankle injuries were enrolled in the study, 20 of them postoperatively received silver hydrogel sheet (SHS) dressing while the other 20 received standard petroleum-based dressings. The results were collected from three-time points sequentially in the 2nd, 6th, and 12th week after surgery. Scar severity was assessed using the Patient and Observer Scar Assessment Scale (PSOAS). In week 2, there was no difference between the test group and the control group. However, from the 6th week, statistically significant differences in the assessment of the scarring process were noticed—both patients and observers assessed the severity of the scar with a significantly lower number of points in the SHS group than in the control group. The scars were shorter, narrower, and their average area was about two times smaller than in the control group.

One of the groups particularly exposed to the toxicity of antibiotics are newborns and infants. In many countries, colloidal silver is given to the child in the first hours of the child’s life to prevent gonococcal conjunctivitis. Shi et al. [92] decided to use a silver dressing in the treatment of an achal anomaly in an infant infected with MRSA. In their case report, they successfully applied a silver dressing, after 14 days the infection was under control and the child was discharged without any further complications.

Chronic wounds are places predisposed to bacterial infection. Disturbed repair processes, often coexisting ischemia, make it impossible for the immune system to effectively fight infections. Therefore, Wang et al. [93] decided to check whether the antibacterial properties of silver can be effectively used in the treatment of patients with such wounds. The patients in the control group were treated with standard methods of chronic wound management, while the study group received a silver alginate dressing. Wounds of patients from the observation group healed significantly faster than the control group (around 25 days in the observation group vs. around 29 days in the control group). They also presented faster granulation tissue growth and wound epithelialization.

Zhang et al. [94] conducted a study in which they explored the effectiveness of thermoplastic polyurethane dressings containing silver nanoparticles (TBU/NS) used in the treatment of open fractures in diabetic patients. TBU/NS dressing was used as a part of postoperative treatment, in the therapy of open fractures of the lower extremities. Patients treated with a traditional dressing showed two times higher risk of developing surgical site infection when compared to the TBU/NS group. This was accompanied by a decrease in the number of white blood cells, C-reactive protein, neutrophils, and the concentration of IL-6 and TNF-alpha.

Hurd et al. [95] presented the results of their retrospective review to find out if treating wounds with an Integrated Care Wound Bundle (ICB) including nanocrystalline silver dressings is more effective than providing wound care without ICB. The outcomes of 2572 patients were analyzed in this study. Wounds treated with the NCS dressing treatment healed significantly faster (10.46 vs. 25.49 weeks). The difference of mean healing time was confirmed for all wound types including diabetic foot ulcer, venous leg ulcer, pressure injuries, surgical wounds, and burns. Despite that, the time between dressing changes was two times higher in the NCS/ICB group (3.98 vs. 1.87 days). Faster wound closure, and the lower number of dressings used resulted in about five times lower mean labor cost in the observation group (6488$ vs. 1251$) [96,97].

**Table 4:** Clinical applications of silver impregnated wound dressings.

Author	Healthy or Diabetic	Type of Wound Dressing	Healing Rate (*p*-Value)	Wound Status	
				Control Wound	Dressed Wound
Meekul et al. [48]	Healthy	Alginate matrix	*p* = 0.057	Saline solution	Alginate Silver Dressing
Hahn et al. [49]	Healthy	Negative-pressure wound therapy	Healing rate was not measured	Negative-pressure wound therapy	Negative-pressure wound therapy + silver
JiHui Chen et al. [51]	Healthy	Moist gauze	*p* < 0.05	Gauze soaked in povidone iodine solution	Silver-containing dressing covered with gauze
Matilda Karlsson et al. [53]	Healthy	Foam or xenograft	*p* < 0.05	Porcine xenograft	Silver-foam dressing
Akin et al. [54]	Healthy	Hydrofiber dressing	Healing rate was not measured	Gauze	Silver-containing hydrofiber dressing
Asgari et al. [89]	Healthy	AgNP based dressing	No statistically significant difference	Hydrocolloid	AgNP dressing
Berard et al. [90]	Healthy	Minimally adherent dressing	No statistically significant difference	Gauze	Minimally adherent silver dressing
Shi et al. [92]	Healthy	Silver sulfate dressing	Infected wound was cured	No control wound	Silver sulfate dressing
Miner et al. [91]	Healthy	Hydrogel sheet dressing	*p* < 0.05	Petroleum-based dressing	Silver hydrogel dressing
Zhang et al. [94]	Diabetic	Thermoplastic polyurethane	*p* < 0.05	Benzalkonium chloride coated dressing	AgNPs with thermoplastic polyurethane
Wang et al. [93]	Healthy	Alginate matrix	*p* < 0.05	Iodoform gauze	Alginate silver dressing
Hurd et al. [95]	Healthy and diabetic	Silver coated polyethylene net	*p* < 0.05	Gauze	Nanocrystaline silver dressing

Much clinical research is still ongoing [96]. Most studies examine the influence of silver biomaterial dressings in dental interventions and burns. This seems to be justified, considering that dental wound healing takes place in an environment that is massively colonized by bacteria, and any dental intervention leads to the transfer of bacteria into the blood. In burns, the risk of infection can be extremely high. For example, when the wound covers more than 40% of the total body surface area, sepsis or other infection complications are responsible for 75% of all death causes in patients [97]. It can be caused by bacteria such as *S. aureus* or *P. aeruginosa*, often characterized by resistance to standard antibiotic therapies, which raises the hope of researchers in the use of newly discovered biomaterials enriched with silver nanoparticles [98].

## 7. Conclusions

Nanoscience is an emerging and significant arena with a wide range of biomedical applications that provide advanced treatment for several kinds of wounds. Nowadays, while antibiotic-resistant microorganisms are some of the main problems to public health, nanoscience is able to offer unique therapeutic approaches in the post-antibiotic era. In the last few years, due to the increase bacterial resistance, traditional treatment is insufficient, and bacterial infection specially in chronic wounds is the major medical problem. Therefore, successful wound healing often demands antimicrobial therapy. The high prevalence of infection together with the increasing risk of antibacterial resistance is the reason why potent alternatives to antibiotics are highly investigated. In those context silver and silver nanoparticles have the ability to simultaneously kill microorganisms and stimulate the regeneration of skin. The unique Ag-NPs properties suggest that they can both effectively prevent wound infections and improve the healing process of damaged tissues in comparison with traditional topical treatments. Of course, as it was mentioned above bacteria are also able to develop resistance mechanisms, reducing the effectiveness of the therapy with silver ions [24]. Luckily, silver presents its antibacterial effect via different mechanisms than standard a-tibiotics. Authors believe that for this reason in the nearest future, most severe infected wounds will be treated with combined therapy of AgNPs and standard antibiotics. The effectiveness of such therapies has been demonstrated in the studies that explored dressings combining silver with colistin or neomycin [68,69]. Several natura or synthetic biomaterials containing silver nanoparticles have already presented promising results in pre-clinical and clinical trials. However, the developed nanosystems and current capabilities in advanced manufacturing, combined with chronic wound knowledge, molecular pathology, and characteristics of phenotype-genotype, have been designed to make the next generation of wound-healing nanoscience. Finally, fabrication of new wound dressing approved by the appropriate medical agency like EDA or FDA, is still a challenge as well. We cannot forget to assess bacteria resistance to any new product that is developed. Thus, there is a continuous demand for improved preparation and analytical techniques that will allow for the translation of nanotechnology-based methods to the clinic.

## Figures and Tables

**Table 1:** Comparision of promising reagents used as wound dressings.

Antibacterial Reagent	Antibacterial Properties	Other Properties	References
AgNPs	gram-positive, gram-negative bacteria	Anti-angiogenic, Anti-inflammatory, Anti-oxidative	[8,10]
ZnO/CuO	gram-positive, gram-negative bacteria	Anti-tumor, anti-oxidative Anti-inflammatory	[15,16]
AuNP	gram-positive, gram-negative bacteria	Anti-inflammatory	[17]
Antimicrobial peptides	gram-positive, gram-negative bacteria	Anti-fungal, anti-viral, anti-parasitic, anti-tumor	[18]
Herbs	gram-positive, gram-negative bacteria	Anti-inflammatory, promoting angiogenesis	[19]

## Data Availability

Not applicable.
